# Increased cerebral vascularization and decreased water exchange across the blood-brain barrier in aquaporin-4 knockout mice

**DOI:** 10.1371/journal.pone.0218415

**Published:** 2019-06-20

**Authors:** Yifan Zhang, Kui Xu, Yuchi Liu, Bernadette O. Erokwu, Pan Zhao, Chris A. Flask, Ciro Ramos-Estebanez, George W. Farr, Joseph C. LaManna, Walter F. Boron, Xin Yu

**Affiliations:** 1 Department of Biomedical Engineering, Case Western Reserve University, Cleveland, OH, United States of America; 2 Department of Radiology, Case Western Reserve University, Cleveland, OH, United States of America; 3 Department of Physiology and Biophysics, Case Western Reserve University, Cleveland, OH, United States of America; 4 Department of Pediatrics, Case Western Reserve University, Cleveland, OH, United States of America; 5 Department of Neurology, Case Western Reserve University, Cleveland, OH, United States of America; 6 Aeromics, LLC, Cleveland, OH, United States of America; Brigham and Women's Faulkner Hospital, UNITED STATES

## Abstract

Aquaporin-4 (AQP4) plays an important role in regulating water exchange across the blood-brain barrier (BBB) and brain-cerebrospinal fluid interface. Studies on AQP-4 knockout mice (AQP4-KO) have reported considerable protection from brain edema induced by acute water intoxication and ischemic stroke, identifying AQP4 as a potential target for therapeutic interventions. However, the long-term effects of chronic AQP4 suppression are yet to be elucidated. In the current study, we evaluated the physiological and structural changes in adult AQP4-KO mice using magnetic resonance imaging (MRI) and immunohistochemical analysis. Water exchange across BBB was assessed by tracking an intravenous bolus injection of oxygen-17 (^17^O) water (H_2_^17^O) using ^17^O-MRI. Cerebral blood flow (CBF) was quantified using arterial spin-labeling (ASL) MRI. Capillary density was determined by immunohistochemical staining for glucose transporter-1 (GLUT1). Compared to wildtype control mice, AQP4-KO mice showed a significant reduction in peak and steady-state H_2_^17^O uptake despite unaltered CBF. Interestingly, a 22% increase in cortical capillary density was observed in AQP4-KO mice. These results suggest that increased cerebral vascularization may be an adaptive response to chronic reduction in water exchange across BBB in AQP4-KO mice.

## Introduction

Water movement across the blood-brain barrier (BBB) and brain-cerebrospinal fluid interface is essential for volume and osmotic regulation in the brain. Aquaporins (AQP) are membrane proteins that allow bidirectional water movement across the phospholipid bilayer of the plasma membrane. Among them, aquaporin-4 (AQP4) is the most highly expressed in the perivascular and subpial astrocytic endfoot membranes of the brain [1,2]. Initial examinations of the AQP4 knockout (AQP4-KO) mice revealed no overt neurological abnormalities or defects in osmoregulation [3]. Further studies reported significant protection from brain edema induced by acute water intoxication and ischemic stroke [4,5]. A recent study also reported reduced infarct volume, cerebral edema, and BBB disruption in AQP4-KO mice after transient focal cerebral ischemia [6]. These studies suggest that AQP4 can be a potential target for therapeutic interventions.

The effects of AQP4 deletion on cerebral structure and physiology have also been investigated. Yao *et al* reported an increase in extracellular volume but unaltered tortuosity in AQP4-KO mice [7]. Saadoun *et al* observed a lack of macromolecule uptake in constitutive AQP4-KO mice, suggesting preserved BBB integrity [8]. Eilert-Olsen and colleagues also reported preserved ultrastructure of capillary endothelial cells, unaltered expression of tight junction proteins, and unaltered vascular permeability to horseradish peroxidase and Evans blue albumin dye [9]. The preserved BBB function was also found in mice with glial-conditional AQP4 deletion [10]. Interestingly, Igarashi *et al* observed an increase in regional cerebral blood flow (CBF) in response to acute AQP4 inhibition [11]. However, baseline CBF in AQP4-KO mice was found to be similar to that in WT mice [6]. These results suggest that chronic adaption to AQP4 deletion have led to the normalization of CBF. However, the mechanisms leading to normalized CBF remain unclear.

The aim of this study was to evaluate the adaptive response to AQP4 deletion in adult AQP4-KO mice. Three quantitative measurements were performed to compare the physiological and structural differences between the AQP4-KO mice and their age-matched WT controls. First, water exchange across BBB was evaluated by tracking an intravenous bolus injection of oxygen-17 (^17^O) enriched water (H_2_^17^O) using magnetic resonance imaging (MRI). Second, cerebral blood flow (CBF) was quantified using arterial-spin-labeling (ASL) MRI. Finally, capillary density was determined by glucose transporter-1 (GLUT1) immunohistochemistry. Our results suggest that increased capillary density may be an adaptive response to chronic reduction in water exchange across BBB in AQP4-KO mice.

## Materials and methods

### Animal preparation

This work was performed in accordance with the Animal Research: Reporting *In Vivo* Experiments (ARRIVE) guidelines. The current study and all the procedures involving animal care/handling were approved by the Animal Care and Use Committee (IACUC) at Case Western Reserve University (Protocol #: 2015–0169).

Two to three months old male AQP4-KO mice (n = 8) and the age-matched wildtype (WT) C57/BL6 mice (n = 6) were characterized. Anesthesia was induced by 3% isoflurane mixed with 100% oxygen, and was maintained with 0.5–1% isoflurane mixed with 30% oxygen and 70% nitrogen. A 30G catheter was inserted to the tail vein for H_2_^17^O injection. For each mouse, 150 μL of 0.9% saline with 12.6% H_2_^17^O enrichment was injected within 25 seconds. Throughout the imaging experiment, the body temperature was maintained at 36–37°C by blowing warm air into the magnet through a feedback control system (SA Instruments, Stony Brook, NY). Respiration rate was maintained at 60–90 breaths/min by adjusting the isoflurane level. Upon the completion of MRI scan, the mouse was euthanized with an overdose of isoflurane and the brain was harvested for immunocytochemistry analyses.

### Imaging methods

MRI studies were performed on a horizontal Biospec 9.4T scanner (Bruker Inc., Billerica, MA, USA). ^1^H images were acquired using a 3-cm birdcage coil (Bruker Inc., Billerica, MA, USA). Multi-slice, T_2_-weighted, axial images were acquired using rapid acquisition with refocused echoes (RARE) sequence.[12] Imaging parameters were: TR/TE, 4000/12 ms; RARE factor, 8; number of averages (NAV), 4; matrix size, 128×128; slice thickness, 3 mm; field of view (FOV), 2×2 cm^2^.

#### ^17^O-MRI

Three-dimensional ^17^O chemical shift imaging (CSI) data were acquired with custom-made, single-loop, 2.5-cm surface coil at a resonance frequency of 54.27 MHz. At baseline, a fully sampled dataset was acquired with 9×9×5 phase-encoding steps covering an FOV of 2×2×1.5 cm^3^ that gave rise to a voxel size of 15 μL. The free induction decays (FIDs) were acquired with 256 data points at 16.8 μs dwell time, leading to a spectral width of 30 kHz. Other imaging parameters were: TR/TE, 12.5/0.64 ms; NAV, 128. Total scan time was 10.8 minutes. Following the acquisition of baseline ^17^O CSI dataset, dynamic ^17^O-CSI data were acquired repeatedly using a keyhole acquisition scheme to improve the temporal resolution. Specifically, 3×3×3 phase-encoding steps in the central k-space were acquired in 337.5 ms with a single average. H_2_^17^O injection was initiated 2 min after the start of dynamic acquisition and lasted for 20 to 25 seconds. A total of 3300 datasets were acquired that covered 2 minutes before and 16.8 minutes after H_2_^17^O injection.

#### Perfusion MRI

Single-slice axial arterial spin-labeling (ASL) images were acquired with a flow-sensitive alternating inversion recovery (FAIR) preparation sequence followed by a centrically encoded fast imaging in steady precession (FISP) readout [13–15]. Specifically, arterial spin labeling was accomplished by slice-selective and non-selective inversion of the magnetization. The inversion pulse used a 3-ms hyperbolic secant adiabatic inversion pulse, and the inversion thickness was set to three times that of the excitation pulse to ensure a uniform inversion over the entire imaging slice. The FISP acquisition was implemented at 1420 ms following the inversion pulse. Imaging parameters were: flip angle, 60°; TR/TE, 2.4/1.2 ms; NAV, 40; matrix size, 128×128; FOV, 3×3 cm^2^; slice thickness, 1.5 mm. A proton density image (M_0_) with no inversion preparation was also acquired using the same imaging parameters. A T_1_ map with the same spatial resolution was acquired with a FISP-based Look-Locker acquisition consisting of a non-selective adiabatic inversion pulse followed by 10 continuous FISP acquisitions with the following parameters: flip angle, 10°; TR/TE, 4.0/2.0 ms; NAV, 40.

### Image processing

All image reconstruction and analysis were performed offline using MATLAB (Natick, MA, USA) based software developed in-house. For the analysis of baseline ^17^O CSI data, the FIDs were applied with 100 Hz line-broadening, zero-padded to 16×16×5, and Fourier transformed to generate the 3D CSI images. For the reconstruction of the dynamic ^17^O data, composite images were generated by replacing the central 3×3×3 k-space of the fully sampled 3D CSI dataset with the dynamic data. Each dynamic dataset used the average of 16 consecutive acquisitions, yielding a temporal resolution of 5.4 s.

**Fig 1:**
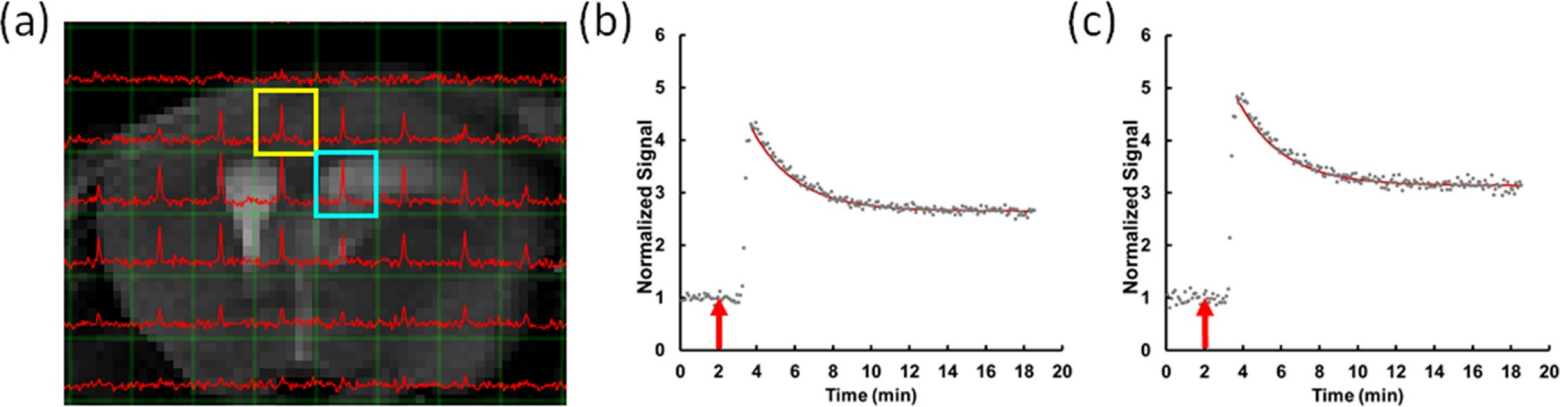
^17^O-MRI of H_2_^17^O uptake and washout. (a) Representative ^17^O-CSI data overlaid on T_2_-weighted ^1^H image. (b,c) Representative time courses of ^17^O signal changes in a CSF voxel (b) and a brain voxel (c), corresponding to the blue and yellow boxes in (a), respectively. Solid dots represent experimental data and red lines the fitted washout curves. Red arrows indicate the start of H_2_^17^O injection.

Once image reconstruction was finished, ^17^O CSI images were overlaid onto the corresponding ^1^H images (Fig 1A). Imaging voxels that overlapped with the brain tissue and the ventricles were selected for signal analysis. The kinetics of H_2_^17^O accumulation and washout in each voxel was quantified by calculating the magnitude of the ^17^O peak in each voxel, normalized by the baseline signal acquired in the initial 2 minutes before H_2_^17^O injection (Fig 1B and 1C). Peak H_2_^17^O uptake was identified as the maximal signal intensity following H_2_^17^O injection. The normalized time course of the H_2_^17^O signal (*SI*) from the peak uptake to the end of the measurement was fit to a mono-exponential function described as

$$SI\left(t\right)=SI_{SS}+\left(SI_{PK}-SI_{SS}\right)e^{-t/\tau }$$

Where *SI*_*PK*_ and *SI*_*SS*_ are the peak and steady-state H_2_^17^O signal, respectively, and τ is the time constant of H_2_^17^O washout.

CBF maps were generated from ASL-MRI data. Briefly, the difference image of the slice-selective and non-selective inversion images (ΔM), the proton density image (M_0_), and the T_1_ maps as described previously [13,14]. Subsequently, CBF was calculated using the model proposed by Pell et al[16], using an arterial T_1_ relaxation time of 2.4 s [17], and a water partition coefficient (λ) of 0.9 [18].

### Immunohistochemistry

After the MRI scan, brain tissue was harvested and fixed with 4% paraformaldehyde for determination of capillary density by immunohistochemistry [19]. Specifically, the paraffin-embedded tissue was sectioned at 5-μm thickness and stained with goat polyclonal primary antibody against glucose transporter-1 (GLUT1, 1:200; Santa Cruz, CA). The stained slices were imaged on a Nikon E600 Eclipse microscope (Nikon Instruments Inc, Melville, NY) with a 20X objective and an Aquos EXi camera (QImaging, Surrey, BC, CA). Capillary density was calculated by counting GLUT1 positive capillaries per unit area of brain tissue using a computer-aided image analysis software ImageJ (NIH, Bethesda, MD).

### Statistical analysis

Acquisition and analyses of the MRI and immunohistochemical data were performed without knowing the genetic background of the animals. Once data analyses were completed, animal identification was obtained, and the mean and standard deviation were calculated for AQP4-KO and WT mice, respectively. Comparison between AQP4-KO and WT mice, as well as between brain tissue and CSF, used two-tailed Student’s *t*-tests. A p value of less than 0.05 was considered statistically significant.

Power analysis was performed to determine the sampled size for the current study. Based on the observed inter-subject variation, it was estimated that a minimum number of 6 mice in each group was needed to detect a 20% increase in H_2_^17^O uptake with 80% power and a significance level of 0.05.

## Results

### Animal characteristics

The age of the mice at the time of MRI scan was 71.0 ± 8.7 days for AQP4-KO mice and 71.2 ± 2.2 days for WT mice. The AQP4-KO mice showed a ~10% increase in body weight as compared to the age-matched WT mice (26.7 ± 2.3 g versus 24.3 ± 1.4 g, p<0.05).

### H_2_^17^O uptake and washout kinetics

**Fig 2:**
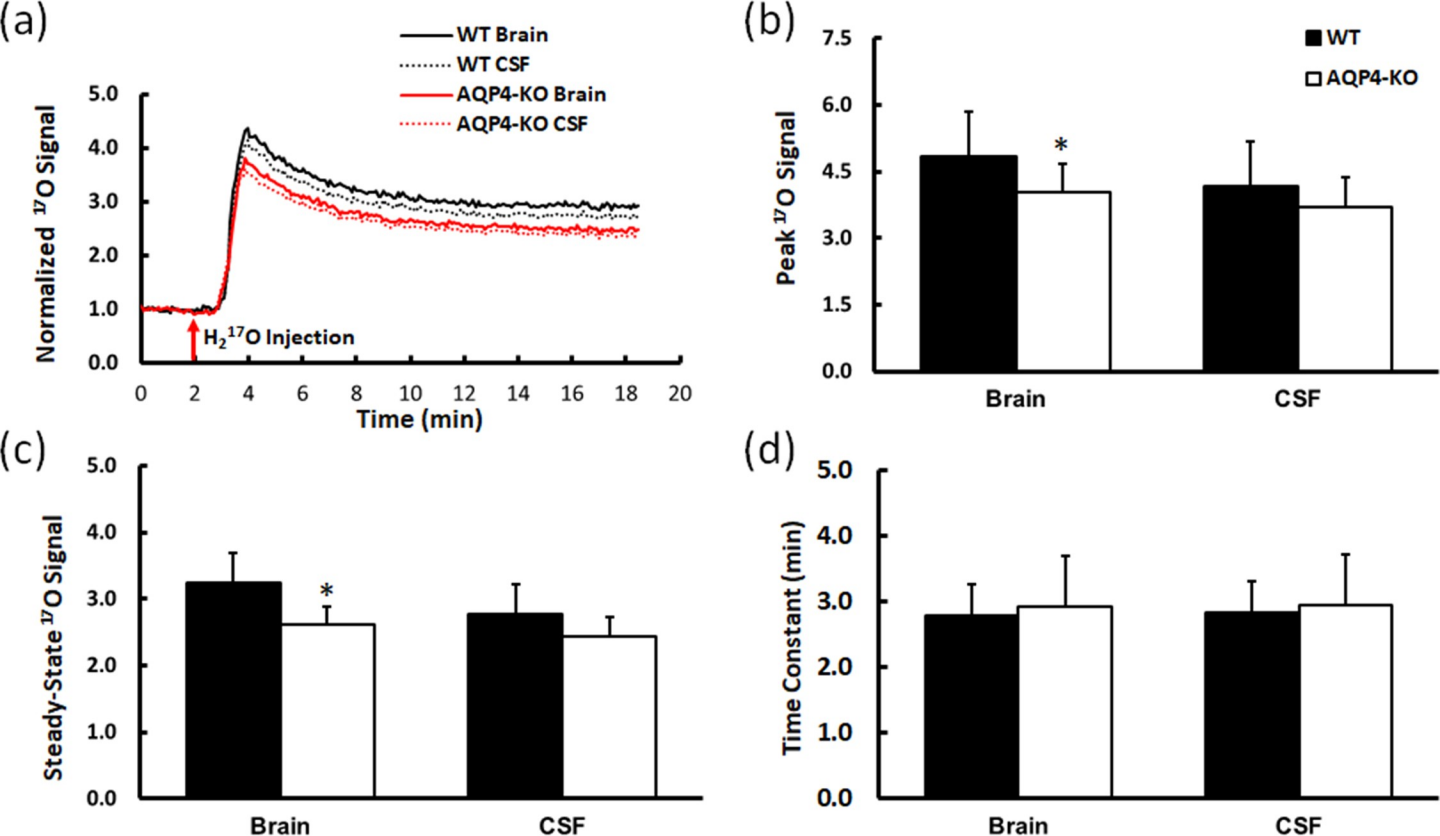
Characteristics of dynamic ^17^O signal. (a) Time courses of group-averaged, baseline-normalized ^17^O signal. (b) Peak H_2_^17^O uptake. (c) Steady-state H_2_^17^O level. (d) Time constant of H_2_^17^O washout. *p<0.05 AQP4-KO versus WT.

Typical time courses of ^17^O signal changes in AQP4-KO and WT mice are shown in Fig 1B and 1C. For each mouse, a total of 35 to 50 voxels in brain and 4 to 8 voxels in CSF were analyzed. Following the injection of H_2_^17^O, both AQP4-KO and WT mice showed a rapid increase in the ^17^O signal that reached peak intensity within 30 seconds. At least 5 data points were collected during this rapid-increase phase. The group-averaged, baseline-normalized time courses of ^17^O signal in brain tissue and cerebrospinal fluid (CSF) are shown in Fig 2A. A monotonically decreasing curve was observed during the washout phase. The signal reached steady-state toward the end of data acquisition.

Compared to that in WT mice, the peak ^17^O signal in AQP4-KO mice was 17% lower (p<0.05, Fig 2B), suggesting reduced H_2_^17^O uptake. AQP4-KO mice also showed a trend of reduced peak ^17^O signal in CSF. However, the difference did not reach statistical significance. The steady-state ^17^O signal showed a similar pattern, with a significant 20% reduction in the brain of AQP4-KO mice (p<0.05, Fig 2C) and a trend of reduction (12%) in the CSF of AQP4-KO mice that did not reach statistical significance. CSF also showed a trend of decreased H_2_^17^O uptake in both WT and AQP4-KO mice comparing to the brain tissue; however, no statistical significance was detected.

The time constant for H_2_^17^O washout is shown in Fig 2D. There was no statistically significant difference in the time constant of H_2_^17^O washout between AQP4-KO and WT mice, either for brain tissue or for CSF. In brain tissue, the time constant was 2.76±0.47 min for WT mice and 2.92±0.76 min for AQP4-KO mice. In CSF, the time constant was 2.82±0.48 and 2.94±0.76 min for WT and AQP4-KO mice, respectively.

### Cerebral perfusion

**Fig 3:**
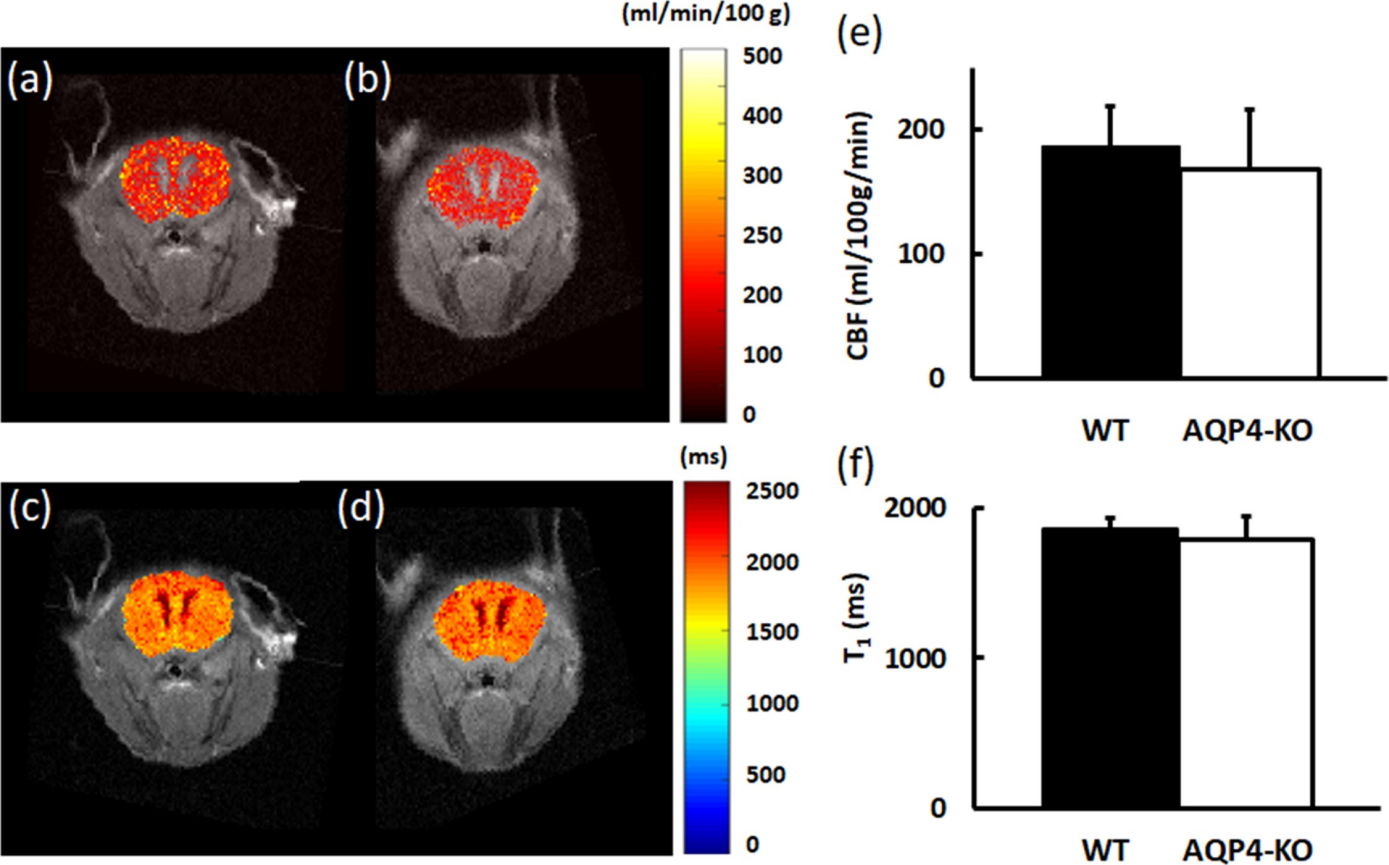
Cerebral perfusion. (a,b) Representative CBF maps of WT and AQP4-KO mice, respectively. (c,d) Representative T_1_ maps of WT and AQP4-KO mice, respectively. (e) Mean cerebral perfusion (excluding ventricles). (f) Mean T_1_ relaxation time (excluding ventricles).

Representative CBF maps from WT and AQP4-KO mice are shown in Fig 3A and 3B, with the corresponding T_1_ maps shown in Fig 3C and 3D. Mean CBF in the entire brain was calculated by excluding the ventricles. Both mean CBF and T_1_ values were similar between the AQP4-KO and WT mice (Fig 3E and 3F). Mean CBF was 186±33 ml/min/100 g in WT mice and 168±47 ml/min/100 g in AQP4-KO mice.

### Capillary density

**Fig 4:**
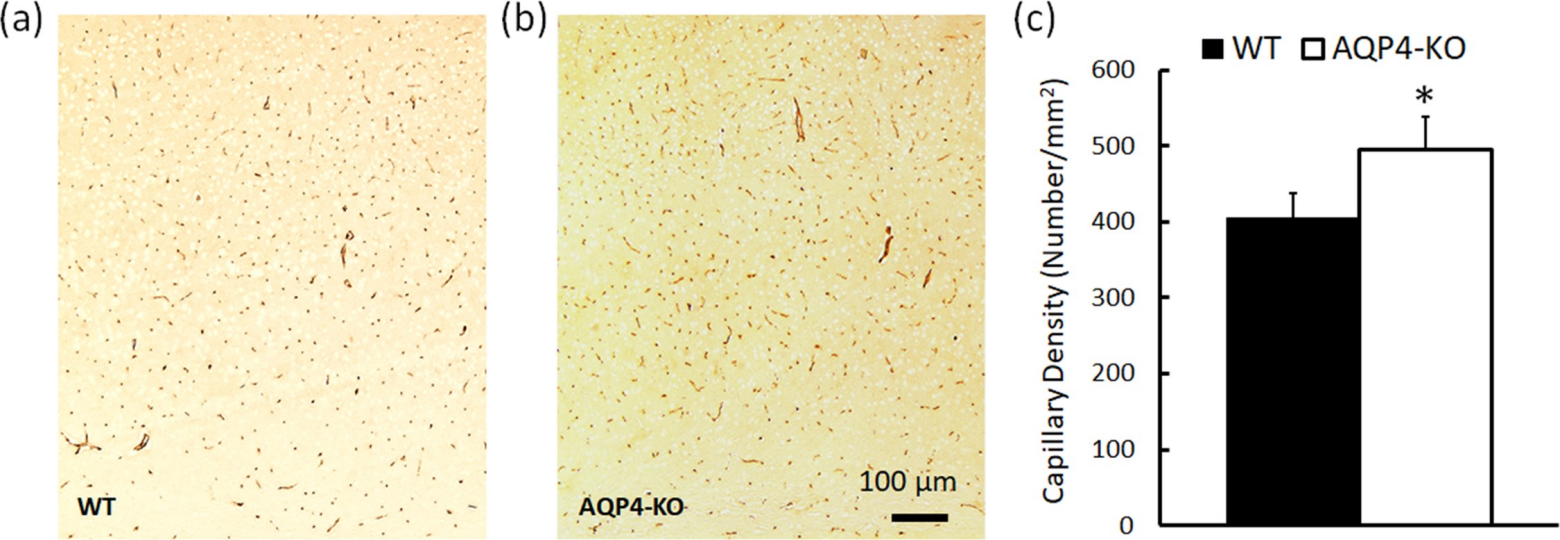
Microvascular density in the cerebral cortex in WT and AQP4-KO mice. (a,b) Representative images of GLUT1 immunohistochemical staining of WT (a) and AQP4-KO (b) mice, respectively. (c) Capillary density (Number/mm^2^) as quantified by the GLUT1 positive staining in brain cortex. *p<0.05 AQP4-KO versus WT.

Fig 4 shows results of GLUT1 staining and capillary density quantified by counting GLUT1 positive capillaries. Compared to WT mice, AQP4-KO mice showed a significant increase in capillary density by about 22%. The number of GLUT1 positive capillaries were 406±32 and 495 ± 45 /mm^2^ in WT and AQP4-KO mice, respectively (P<0.05).

## Discussion

In this study, we characterized the sequestration and subsequent washout of H_2_^17^O in the brain of AQP4-KO mice by directly observing kinetic changes in the ^17^O signal after an intravenous bolus injection of H_2_^17^O. By using a keyhole-based data acquisition scheme, a 5.4-s temporal resolution was achieved that enabled the delineation of the rapid signal increase immediately after H_2_^17^O injection. Consistent with a previous study using T_2_-weighted proton MRI for indirect H_2_^17^O detection [20], our results also showed decreased H_2_^17^O uptake and retention in AQP4-KO mice. This reduction in H_2_^17^O uptake is a direct consequence of the lack of AQP4-mediated water movement across the BBB. It is also consistent with a previous study that reported reduced BBB water uptake in mice with glial-conditional AQP4 deletion [10].

It is worth noting that CSF also showed a trend of reduced H_2_^17^O uptake in AQP4-KO mice, although no statistical difference was detected in the current study. Previously, Igarashi *et al* evaluated H_2_^17^O uptake in AQP4-KO mice indirectly by T_2_-weighted proton MRI. They observed decreased H_2_^17^O uptake into the third ventricle of AQP4-KO mice [20]. A possible explanation for this discrepancy could be the low spatial resolution in our current study. Due to the low sensitivity of ^17^O-MRI, a keyhole-based method was used to capture the kinetics of ^17^O signal. Since only the center of the k-space was updated during dynamic data acquisition, the dynamic information is only of low spatial resolution [21], which may limit the ability of this methods to detect changes in small regions such as CSF. Future improvement could be made to improve the spatial resolution by using other fast imaging methods such as ultra-short echo-time (UTE) and compressed sensing.

Despite reduced H_2_^17^O uptake by AQP4-KO mice, the time constant of H_2_^17^O washout was similar between AQP4-KO and WT mice. Previous studies using oxygen-15 labeled water (H_2_^15^O) and positron emission tomography (PET) have demonstrated that the kinetics of H_2_^15^O is dominated by cerebral perfusion [22,23]. Like H_2_^15^O, the washout kinetics of H_2_^17^O is also primarily governed by cerebral perfusion, with only a slight contribution from water movement across the BBB. Thus, the lack of difference in the kinetics of H_2_^17^O washout suggests that cerebral perfusion was similar in AQP4-KO and WT mice. Indeed, CBF measured by ASL-MRI in the current study was similar between AQP4-KO and WT mice. A recent study employing a multi-echo ASL technique also reported unaltered CBF in AQP4-KO mice, although the exchange time of labeled intravascular water across BBB was significantly increased by 31% [24]. Similar CBF between AQP4-KO and WT mice was also observed by Yao *et al* using laser Doppler flowmetry [6]. On the other hand, a previous study has reported that acute inhibition of AQP4 by the putative AQP4 inhibitor TGN-020 induced a significant increase in CBF in WT mice [11]. Hence, the lack of change in baseline CBF suggests that chronic AQP4 deletion can lead to an adaptive response toward normalized cerebral perfusion.

A major finding of the current study is the increase in capillary density in AQP4-KO mice assessed by GLUT1 immunocytochemistry. Comparing to other methods of assessing vessel density, e.g. fluorescent microscopy [25], GLUT1 immunocytochemistry is specific to micro vessels with blood-brain barrier but not to arterioles [26]. With unaltered CBF, this increase in capillary density will lead to increased mean transit time (MTT = cerebral blood volume/CBF) in AQP4-KO mice. Hence, it is possible that chronic AQP4 deletion may have led to the adaptive response to compensate for reduced water exchange at BBB by increasing the time for exchange via increased microvasculature. Another hypothesis that is worth investigating is the role of AQP4 in facilitating oxygen diffusion. The central pore in an AQP4 tetramer has been shown to be permeable to gases such as oxygen, carbon dioxide, nitric oxide, and ammonia [27–29]. This newly identified role of AQP4 suggests that AQP4 deletion may lead to insufficient oxygen supply when metabolic demand is high. Indeed, Thrane *et al* have reported impaired oxygenation in areas remote from brain microvessels in AQP4-KO mice during cortical spreading depression, suggesting the involvement of AQP4 in oxygen diffusion *in vivo* [30]. Hence, the increase in capillaries may also be in response to reduced oxygen diffusion associated with AQP4 deletion. Further investigation of cerebral metabolic activities in AQP4-KO mice will allow new insights into the role of AQP4 in oxygen delivery and regulating cerebral metabolism.

Our current study used H_2_^17^O injected via tail vein as a tracer. While the results suggest that ^17^O-MRI is sensitive to altered water exchange across BBB, it should be noted that our current study does not provide quantitative assessment of BBB permeability to water. Tail vein injection causes a dispersion of the arterial input function, which needs to be measured experimentally in order to quantify BBB permeability through kinetic modeling. Due to the low-resolution of ^17^O-MRI, dynamic changes of ^17^O signal in carotid artery cannot be reliably measured with the current method. A feasible approach to obtain the arterial input function would be to measure ^17^O signal in ventricular cavity using a two-coil system and interleaved acquisition. Alternatively, intra-carotid injection allows the tracking of the first pass of tracer, which can provide reliable measurement of the permeability-surface area product, as was demonstrated in PET studies using H_2_^15^O as a tracer [31]. This approach requires further improvement in temporal resolution. Future development that combines compressed sensing with UTE may enable quantitative assessment of BBB permeability by ^17^O-MRI [32].

In conclusion, we observed increased capillary densities and reduced water exchange across BBB in AQP4-KO mice. These findings suggest an adaptive response to chronic AQP4 deletion toward increased MTT for increased time of water exchange and perhaps oxygen diffusion as well across BBB. Future work on oxygen delivery and cerebral metabolism in AQP4-KO mice is warranted to elucidate the role of AQP4 as a gas channel in addition to a water channel.
